# Relationship of Transportation Noise and Annoyance for Two Metropolitan Cities in Korea: Population Based Study

**DOI:** 10.1371/journal.pone.0169035

**Published:** 2016-12-22

**Authors:** Joo Hyun Sung, Jiho Lee, Sang Jin Park, Chang Sun Sim

**Affiliations:** Department of Occupational and Environmental Medicine, Ulsan University Hospital, University of Ulsan College of Medicine, Ulsan, Rep of Korea; West Virginia University, UNITED STATES

## Abstract

Transportation noise is known to have negative impact on both public health and life quality. This study evaluated the relationship between transportation noise and annoyance levels, and also the difference of annoyance levels in two metropolitan cities based on epidemiologic surveys. Two thousand adult subjects living in Seoul and Ulsan were enrolled by stratified random sampling on the basis of noise maps from July 2015 to January 2016. Individual annoyance in accordance with transportation noise levels in two metropolitan cities were surveyed using an 11-point visual analog scale questionnaire. The results show that transportation noise level was significantly correlated with annoyance in both cities. Logistic regression analysis revealed that the risk of being ‘highly annoyed’ increased with noise level (L_dn_, day-night average sound level) in both cities. After adjusting for age, residence period, sociodemographic factors (sex, education, marriage, income, alcohol, smoking, and exercise) and noise sensitivity, the risk of being ‘highly annoyed’ was increased with noise levels in both cities. In comparison to those of areas with noise levels below 55 dBA, the adjusted odds ratios of ‘highly annoyed’ for areas with 55–65 dBA and over 65 dBA were 2.056 (95% confidence interval [CI] 1.225–3.450), 3.519 (95% CI 1.982–6.246) in Seoul and 1.022 (95% CI 0.585–1.785), 1.704 (95% CI 1.005–2.889) in Ulsan, respectively. Based on the results of a population study, we showed that transportation noise levels were significantly associated with annoyance in adults. However, there were some differences between the two cities. In this study, there were differences in transportation noise between the two cities. Seoul has complex noise (traffic and aircraft), compared to single road traffic noise in Ulsan. Therefore, single and complex transportation noise may have different effects on annoyance levels.

## Introduction

Noise refers to unwanted sound and environmental noise is defined as noise from all sources except for industrial workplaces [1]. Previously, the main concern of noise problems was occupational exposure to noise in industrial workplaces. However, with increasing urban population density due to urbanization and industrialization [2] noise has become an environmental pollutant to which we are constantly exposed in our everyday lives. There has been a growing interest in environmental noise exposure [3] such as transportation noise (cars, trains and aircraft), neighborhood noise, and leisure noise [4].

Because environmental noise levels are typically lower than those found in industrial workplaces, there had previously been relatively few studies on environmental noise. During the past 3 decades, growing interest in the potential adverse health effects of environmental noise has resulted in a significant increase of additional studies. Furthermore, the World Health Organization (WHO) reported levels of environmental noise associated with individual annoyance and sleep disturbance in 2002 and 2009, respectively [5, 6], and reported comprehensively on the health effects of environmental noise in their 2011 "Burden of Disease from Environmental Noise" [1].

Since the publication of these WHO reports, numerous studies have reported the effects of environmental noise on health, including hearing impairment [7, 8], tinnitus [9], cardiovascular disease [10, 11], cognitive impairment [12], sleep disturbance [13] and annoyance [5].

Among these, annoyance is a major health effect of environmental noise exposure. Since it was first introduced by Schultz in 1978 [14], this concept has been widely used in the assessment of the health effects of environmental noise. Annoyance is converted into a 100-point scale, with scores of 50 points or more and 72 points or more defined as ‘annoyed’ and ‘highly annoyed’ respectively. Annoyance has a dose-response relationship with noise exposure and threshold levels associated with annoyance have also been proposed [15]. For these reasons, annoyance levels are widely used as assessment tools for evaluating the health effects of environmental noise exposure at levels lower than those in industrial workplaces.

Most recent domestic studies on the health effects of noise have been conducted in small populations or were experimental studies. Furthermore, large-scale studies assessing the health effects of environmental noise in other countries are also scarce. Therefore, the present study evaluated the relationship between the degree of annoyance and actual levels of transportation noise based on noise map in a general population.

## Materials and Methods

### Study population

**Fig 1:**
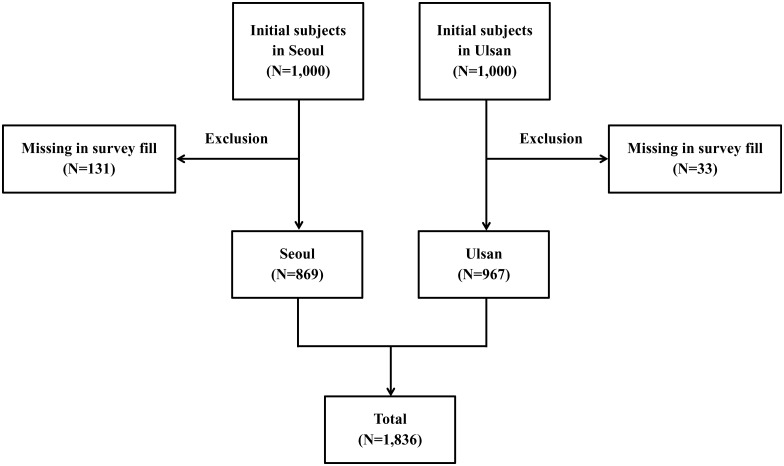
Scheme of selection criteria of subjects.

This study was approved by the Institutional Review Board of Ulsan University Hospital (IRB No. 2014-08-008). All subjects participated voluntarily and approved written informed consent. A total of 1,000 subjects each in Yangcheon-gu, Seoul and Nam-gu, Ulsan finally agreed to participate in this study. The subjects were stratified according to noise level exposure based on noise map data; they approved examination agreements and informed consent. Yangcheon-gu, Seoul is an area that is exposed to both aircraft and road traffic noise, while Nam-gu, Ulsan is characteristically exposed to road traffic noise alone. Survey researchers visited each subject’s home and conducted the survey from July 2015 to January 2016. Of 2,000 subjects, 1,836 subjects, except for 164 subjects with missing survey results (131 in Yangcheon-gu, Seoul and 33 in Nam-gu, Ulsan), were finally included in this study (Fig 1).

### Survey

The questionnaire contained questions regarding sociodemographic variables such as age, sex, education, marital status, income, smoking status, alcohol, and exercise and residence period. Education level was divided into high school graduate or below and college graduate or above, and marital status was divided into married, single, or other (bereavement, divorce, separation, cohabitation). Average monthly income was divided into less than KRW (Korean won) 3 million and KRW 3 million or more. Smoking status was divided into current smoker and current non-smoker (ex-smoker and non-smoker); current smoker was defined as having a history of smoking more than 100 cigarettes in one’s life and continuing smoking [16]. Drinking status was divided into current drinker and current non-drinker, and exercise was divided into current regular exercise and non-regular exercise.

Sensitivity to noise and annoyance due to transportation noise were assessed using an 11-point visual analog scale (VAS) which was developed based on International Organization for Standardization Technical Specification (ISO/TS) 15666 (2003) [17]. In the present study, annoyance due to transportation noise was assessed using a 0–10 point scale; subjects with 72% or more of point scale (8–10 points) were classified as ‘highly annoyed’, while 50% or more of point scale (6–10 points) were classified as ‘annoyed’.

### Transportation noise levels

In order to estimate noise levels from each subject’s residential environment, this study used three-dimensional noise maps created in 2014. Average noise level was calculated using Noise Production Program (Cadna A, DataKustik, Germany) at the facade of the residential buildings of subjects based on the address. The noise indicator used in this study is day-night equivalent sound level (L_dn_). L_dn_ is defined as average sound level during daytime (07:00–22:00) and night time (22:00–07:00), and night time gets a penalty of 10 dB [5]. Noise levels were classified as less than 55 dBA, 55–65 dBA, and more than 65 dBA.

### Statistical analysis

Analysis of variance (ANOVA) was used to compare age, residence period, and noise sensitivity according to noise levels. Chi-square tests were used to compare gender, education, marital status, income, history of smoking, history of drinking, regular exercise, highly annoyed, and annoyed according to noise levels.

In order to calculate the odds ratios (OR) of being annoyed and highly annoyed according to noise level, logistic regression analysis was used to compare to subjects who were exposed to noise levels below 55 dBA. In addition, adjusted odds ratios (aOR) were calculated using multiple logistic regression to adjust for confounding variables that could potentially affect annoyance, including age, residence period, gender, education, marital status, average monthly income, smoking status, drinking status, exercise, and sensitivity.

All data were analyzed using IBM SPSS Statistics for Windows, version 21.0 (IBM SPSS Inc., Chicago, IL), and *p*-values of less than 0.05 were considered statistically significant.

## Results

The present study included a total of 1,836 individuals, 967 (52.7%) and 869 (47.3%) of whom resided in Nam-gu, Ulsan and Yangcheon-gu, Seoul, respectively. The average age of those exposed to 55–65 dBA was younger than other group and the average residence period of those exposed to over 65 dBA was shorter than other group. But there were no significant difference. As noise levels increased, the rate of married subjects tended to be greater (*p* = 0.002) and monthly income tended to be higher (*p*<0.001). The rate of current smokers was the highest among those exposed to less than 55 dBA, the lowest noise level (*p*<0.001); the rate of those who regularly exercised was also the highest in this group (*p*<0.001). There were no significant differences in other variables (Table 1).

**Table 1:** General characteristics of subjects according to noise levels.

Variables		Under 55 dBA (n = 993)	55–65 dBA (n = 593)	Over 65 dBA (n = 411)	*p*-value
Age (years)		47.1±16.7	46.8±16.0	47.2±14.9	0.927
Residence period (years)		9.3±9.1	9.2±8.4	8.5±7.1	0.288
Noise sensitivity		5.2±2.3	5.2±2.2	5.4±2.1	0.523
Sex	Men	348 (39.2)	211 (38.0)	137 (34.8)	0.315
	Women	539 (60.8)	344 (62.0)	257 (65.2)	
Education level	High school and less	432 (48.7)	248 (44.7)	178 (45.2)	0.259
	College and more	455 (51.3)	307 (55.3)	216 (54.8)	
Marital status	Single	255 (28.7)	150 (27.0)	90 (22.8)	0.002
	Married	495 (55.8)	346 (62.3)	262 (66.5)	
	Etc.*	137 (15.4)	59 (10.6)	42 (10.7)	
Monthly income (1,000 KRW)	< 3,000	420 (47.4)	203 (36.6)	106 (26.9)	<0.001
	≥ 3,000	467 (52.6)	352 (63.4)	288 (73.1)	
Smoking status	Non smoker	734 (82.8)	503 (90.6)	354 (89.8)	<0.001
	Smoker	153 (17.2)	52 (9.4)	40 (10.2)	
Alcohol status	No drink	450 (50.7)	299 (53.9)	185 (47.0)	0.109
	Drink	437 (49.3)	256 (46.1)	209 (53.0)	
Regular exercise	No	282 (31.8)	242 (43.6)	167 (42.4)	<0.001
	Yes	605 (68.2)	313 (56.4)	227 (57.6)	

Unit, mean±standard deviation, number (percentage)

*p*-value was calculated by ANOVA for continuous variable and chi-square test for categorical variable

*Etc.: bereavement, divorce, separation, cohabitation

**Fig 2:**
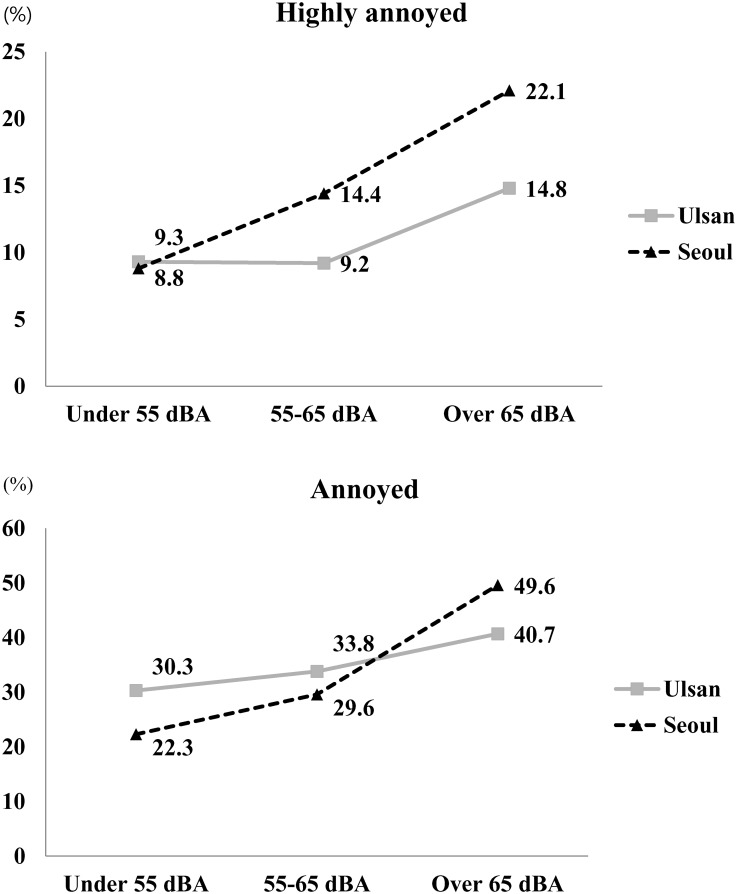
Proportion of ‘highly annoyed’ and ‘annoyed’ in two metropolitan cities according to noise levels.

Distribution of ‘highly annoyed’ and ‘annoyed’ population according to noise exposure levels are given in Table 2. Overall, the percentage of highly annoyed subjects tended to increase with higher levels of noise exposure, from 9.0% of subjects in the less than 55 dBA group, to 11.5% and 17.3% in the 55–65 dBA and greater than 65 dBA groups, respectively (*p*<0.001). The percentage of annoyed subjects also tended to increase, from 29.5% among those exposed to less than 55 dBA to 31.9% and 43.7% the 55–65 and greater than 65 dBA groups, respectively (*p*<0.001). By residential area, the percentage of highly annoyed subjects in Ulsan was similar between the less than 55 dBA and 55–65 dBA groups (9.3% and 9.2%, respectively), but the percentage was higher (14.8%) among those exposed to more than 65 dBA (*p* = 0.044). The percentage of annoyed subjects was also similar between the less than 55 dBA and 55–65 dBA groups (30.3% and 33.8%, respectively), but higher (40.7%) in the group of subjects exposed to more than 65 dBA (*p*<0.001). In Seoul, the percentage of highly annoyed subjects tended to increase with noise level, from 8.3% in the less than 55 dBA group to 14.4% and 21.1% in the 55–65 dBA and more than 65 dBA groups, respectively (*p* < 0.001). The percentage of annoyed subjects also tended to increase with increasing noise level, from the 22.3% in less than 55 dBA group, to 29.6% and 49.6% in the 55–65 dBA and more than 65 dBA groups, respectively (*p*<0.001, Table 2, Fig 2).

**Table 2:** Distribution of “highly annoyed” and “annoyed” population according to noise exposure level.

		Under 55 dBA	55–65 dBA	Over 65 dBA	*p*-value
Total	Highly annoyed	80 (9.0)	64 (11.5)	68 (17.3)	<0.001
	Annoyed	230 (25.9)	177 (31.9)	172 (43.7)	<0.001
Seoul	Highly annoyed	43 (8.8)	36 (14.4)	29 (22.1)	<0.001
	Annoyed	109 (22.3)	74 (29.6)	65 (49.6)	<0.001
Ulsan	Highly annoyed	37 (9.3)	28 (9.2)	39 (14.8)	0.044
	Annoyed	121 (30.3)	103 (33.8)	107 (40.7)	0.022

Unit, number (percentage)

*p*-value was calculated by chi-square test

The results of the analysis of risk of annoyance due to noise are shown in Table 3. For all subjects, the ORs of being highly annoyed in the 55–65 dBA and more than 65 dBA groups compared to the less than 55 dBA groups were 1.315 (95% CI 0.929–1.861) and 2.104 (95% CI 1.486–2.980), respectively. After adjusting for age, residential period, social economic factors (sex, education, marital status, income, alcohol, smoking, and exercise) and noise sensitivity, the aORs of being highly annoyed in the 55–65 dBA and more than 65 dBA groups were 1.483 (95% CI 1.020–2.157) and 2.362 (95% CI 1.615–3.453), respectively, while the aORs of being annoyed in these groups were 1.453 (95% CI 1.124–1.879) and 2.405 (95% CI 1.825–3.169), respectively.

**Table 3:** Odds ratio and adjusted odds ratio of “highly annoyed” and “annoyed” according to noise exposure level.

		Noise exposure (L_dn_)	Number	OR ^a^	95% CI	aOR ^b^	95% CI
Total	Highly annoyed	Under 55 dBA	887	1.000		1.000	
		55–65 dBA	555	1.315	0.929–1.861	1.483	1.020–2.157
		Over 65 dBA	394	2.104	1.486–2.980	2.362	1.615–3.453
	Annoyed	Under 55 dBA	887	1.000		1.000	
		55–65 dBA	555	1.338	1.059–1.689	1.453	1.124–1.879
		Over 65 dBA	394	2.213	1.725–2.840	2.405	1.825–3.169
Seoul	Highly annoyed	Under 55 dBA	488	1.000		1.000	
		55–65 dBA	250	1.741	1.086–2.791	2.056	1.225–3.450
		Over 65 dBA	131	2.942	1.753–4.938	3.519	1.982–6.246
	Annoyed	Under 55 dBA	488	1.000		1.000	
		55–65 dBA	250	1.462	1.035–2.065	1.603	1.097–2.342
		Over 65 dBA	131	3.424	2.288–5.126	3.870	2.482–6.036
Ulsan	Highly annoyed	Under 55 dBA	399	1.000		1.000	
		55–65 dBA	305	0.989	0.591–1.656	1.022	0.585–1.785
		Over 65 dBA	263	1.703	1.054–2.752	1.704	1.005–2.889
	Annoyed	Under 55 dBA	399	1.000		1.000	
		55–65 dBA	305	1.172	0.851–1.612	1.304	0.911–1.866
		Over 65 dBA	263	1.576	1.138–2.183	1.801	1.245–2.606

^a^ Odds ratio was calculated by logistic regression analysis.

^b^ Adjusted odds ratio was calculated by multiple logistic regression analysis after adjusting age, residence period, social economic factor (sex, education, marital status, income, alcohol, smoking, exercise) and noise sensitivity

In Ulsan, the ORs of being highly annoyed in the 55–65 dBA and more than 65 dBA groups were 0.989 (95% CI 0.591–1.656) and 1.703 (95% CI 1.054–2.752), respectively, while the ORs of being annoyed were 1.172 (95% CI 0.851–1.612) and 1.576 (95% CI 1.138–2.183), respectively. In addition, the aORs of being highly annoyed in 55–65 dBA and more than 65dBA groups were 1.022 (95% CI 0.585–1.785) and 1.704 (95% CI 1.005–2.889), respectively, while the aORs of being annoyed in the 55–65 dBA and more than 65 dBA groups were 1.304 (95% CI 0.911–1.866) and 1.801 (95% CI 1.245–2.606), respectively. In Seoul, the ORs of being highly annoyed in the 55–65 dBA and more than 65 dBA groups were 1.741 (95% CI 1.086–2.791) and 2.942 (95% CI 1.753–4.938), respectively, while the ORs of being annoyed were 1.462 (95% CI 1.035–2.065) and 3.424 (95% CI 2.288–5.126). In addition, the aORs of being highly annoyed in the 55–65 dBA and more than 65 dBA groups were 2.056 (95% CI 1.225–3.450) and 3.519 (95% CI 1.982–6.246), respectively, while the aORs of being annoyed were 1.603 (95% CI 1.097–2.342) and 3.870 (95% CI 2.482–6.036), respectively (Table 3).

## Discussions

In order to assess the health effects of environmental noise, the present study compared annoyance levels according to transportation noise levels between subjects living in Nam-gu, Ulsan who are exposed to road traffic noise alone, and those living in Yangcheon-gu, Seoul who are exposed to both road traffic noise and aircraft noise. Noise annoyance is defined as an unpleasant feeling caused by noise [18]. Noise annoyance shows dose-response relationships from a relatively low noise level (about 50 dBA), however the degree of annoyance varies according to the type of transportation noise at the same noise levels [5].

In Ulsan with road traffic noise alone, compared to the 55 dBA noise level group, both the ORs and aORs of being highly annoyed and annoyed were significantly higher among individuals exposed to noise levels above 65 dBA. However, in Seoul, both the ORs and aORs of being highly annoyed and annoyed increased with increasing noise levels, showing dose-response relationships. In addition, the ORs and aORs of being highly annoyed and annoyed were higher than those of Ulsan. Despite sampling from populations exposed to the same noise levels based on noise map information, annoyance levels in subjects exposed to both road traffic and aircraft noise increased much more with increasing noise levels compared to those exposed to road traffic noise alone.

Studies before 2,000 reported that, at the same noise levels, aircraft noise had the most significant impact on annoyance, followed by road traffic noise, and that train noise had relatively less impact on annoyance levels. Since 2,000, there have been many studies on single noise, including traffic, aircraft, and train noise [19]. However, studies of health effects on complex transportation noise exposure are scarce [5, 15] and the results have shown inconsistent [4].

The current investigation reveals that noise level correlate with human health; however, sound characteristics are a less well-known factor affecting human health [20–22]. Even at the same noise levels, the sound frequencies of road traffic and aircraft noise differ [23], and the results of a previous study indicated that noise threshold levels associated with health effects might differ according to the frequency of sound [24]. In addition, road traffic noise is almost continuous, whereas aircraft noise is characteristically discontinuous because it occurs mainly when aircraft is taking off or landing [25, 26]. Thus, differences in sound characteristics in addition to noise levels may also affect annoyance [27, 28], and these features may be associated with increased annoyance even at the same noise levels as observed in the present study.

The present study has some limitations. First, because this was a cross-sectional study, it was not possible to analyze the effect of noise on annoyance as causal relationship. Second, annoyance was measured based on a survey as a subjective indicator in this study. Since the noise levels in the present study were relatively low compared with those in industrial workplaces and appropriate methods for objectively assessing annoyance are not yet available, various biases were likely to occur during the survey process. Third, sound characteristics were not analyzed. Assessment of sound characteristics could offer a better understanding of which sound characteristics in addition to noise levels might affect annoyance.

Nevertheless, the results of the present study have several important implications. First, to our knowledge, this study is the first to assess the health effects of environmental noise on annoyance in a large-scale, population-based study in South Korea. The results of this study offer data to directly compare the effects of environmental noise in South Korea to effects reported in other countries. Second, although a number of previous studies have compared the effects of single noises such as road traffic, aircraft, and train noise (primarily road traffic noise) [19], the present study is significant in that it also considered real-life exposure to complex traffic noise relative to the effects of single environmental noises.

## Conclusions

The results of this population-based study indicate that exposure to complex noise such as road traffic and aircraft noise is associated with increased annoyance levels compared to annoyance levels to noise exposure to road traffic noise alone, even at similar noise levels. These results remained consistent after controlling for various variables that could affect subjective judgments. We think that the reason of these differences was associated with sound characteristics of complex noise and single noise. However, there is few study considering sound characteristics in evaluating health effects of environmental noise. So we recommend that future studies on the health effects of environmental noise should consider sound characteristics and the type of sound sources as well as noise level.

## Supporting Information

S1 Dataset Full dataset of tables. Supplement file.xlsx.
(DOCX)

S1 File Supplementary information for dataset. Supporting information for dataset.docx.
(XLSX)
