# Supplementary material for: Relationship of Transportation Noise and Annoyance for Two Metropolitan Cities in Korea: Population Based Study
Source: PLoS One. 2016 Dec 22;11(12):e0169035. doi: 10.1371/journal.pone.0169035 (PMC5179245; doi:10.1371/journal.pone.0169035)
Supplement: S1 Dataset — Supplement file.xlsx. (DOCX) [file pone.0169035.s001.docx]

This file provides contextual information for data file linked to the publication: Sung JH, Lee J, Park SJ, Sim CS. Relationship of transportation noise and annoyance for two metropolitan cities in Korea: population based study.

We described the all possible data used in the manuscript in a spreadsheet.

There is single excel file. Data file “Supplement file” consists of summary data of our study. The file contains the following columns:

ID: Code indicating the ID of subjects

Region: Code indicating the region of residential of subjects

Noise: Code indicating the noise exposure level (L_dn_). “Under 55” means that the noise exposure level is under 55 dBA. “55~65” means that the noise exposure level is between 55 and 65 dBA. “Over 65” means that the noise exposure level is over 65 dBA.

Age: Code indicating the age of subject at examination day.

Residence: Code indicating the period of residence in that dwelling of subjects.

Noise_sensitivity: Code indicating noise sensitivity of subjects. This scale was visual analog scale ranged from 0 to 10. “0” means least sensitivity and “10” means most sensitivity.

Sex: Code indicating sex.

Education: Code indicating education level of subjects.

Marital_status: Code indicating marital status of subjects. “married” means married, “single” means not married, and “etc” means bereavement, divorce, separation, cohabitation at examination day.

Income: Code indicating average monthly income of subjects at examination day.

Smoking: Code indicating smoking status of subjects.

Alcohol: Code indicating drinking status of subjects. “yes” means current drinker, “no” means current non-drinker.

Exercise: Code indicating exercise status of subjects. “yes” means current regular exercise, “no” means current non-regular exercise.

Annoyance: Code indicating noise annoyance of subjects. This scale was visual analog scale ranged from 0 to 10. “0” means least annoyed and “10” means most annoyed.

HA: Code indicating “highly annoyed” of subjects. “normal” means that score ranged between 0 and 7, “abnormal” means that score ranged between 8 and 10.

A: Code indicating “annoyed” of subjects. “normal” means that score ranged between 0 and 5, “abnormal” means that score ranged between 6 and 10.
